# Persons with multiple sclerosis older than 55 years: an analysis from the German MS registry

**DOI:** 10.1007/s00415-024-12286-4

**Published:** 2024-03-22

**Authors:** Yasemin Goereci, David Ellenberger, Paulus Rommer, Veronika Dunkl, Heidrun Golla, Uwe Zettl, Alexander Stahmann, Clemens Warnke

**Affiliations:** 1 Department of Neurology, Faculty of Medicine and University Hospital Cologne, University of Cologne, Kerpener Str. 62, 50937 Cologne, Germany; 2 German MS Register by the German MS Society, MS Research and Project Development gGmbH [MSFP], Hannover, Germany; 3 Department of Neurology, Neuroimmunological Section, University of Rostock, Rostock, Germany; 4 Department of Neurology, Medical University of Vienna, Vienna, Austria; 5 Department of Palliative Medicine, Faculty of Medicine and University Hospital Cologne, University of Cologne, Cologne, Germany

**Keywords:** Epidemiology, Multiple sclerosis, Disease modifying therapy, Age

## Abstract

**Background:**

Persons with MS (PwMS) ≥ 55 years are underrepresented in therapy studies leading to a lack of evidence.

**Objective and methods:**

To study the subgroup of PwMS ≥ 55 years in the German MS registry in comparison with PwMS < 55 years. Endpoints of interest were the grade of disability, leading symptoms, clinical and magnetic resonance imaging activity, and use of disease modifying therapy.

**Results:**

At the time of analysis, data from 40,428 PwMS were available for analysis. In PwMS aged ≥ 65 and PwMS aged ≥ 55 to 64 years, compared with PwMS aged < 55 years, the mean Expanded Disability Status Scale Scores were higher (5.3, 4.2 and 2.7, respectively), while the proportion of individuals with current use of disease modifying therapy was lower (42.6%, 60.9% and 76.7%, respectively). The older patient groups were more likely to be labeled with progressive MS and the frequency of occupational invalidity was high (38.8% in PwMS aged ≥ 55 to 64 years). Gait disorder, fatigue, bladder dysfunction, and spasticity were among the leading symptoms in PwMS aged ≥ 55 years.

**Conclusion:**

PwMS ≥ 55 years have a high degree of disability, but a large proportion do not receive disease modifying therapy, exposing an unmet need.

## Introduction

The absolute and relative number of persons with multiple sclerosis (PwMS) above the age of 55 years is increasing [1]. Recently published health insurance data from Germany demonstrated an overall increase of MS prevalence and showed that the mean age of PwMS rose between 2010 and 2017, independent of the disease course classification [2]. The aging of the general population, improved treatment options for MS, and more sensitive MS diagnostic criteria might contribute to these observations [3].

Importantly, clinical trials relevant for approval of disease-modifying therapies (DMT) rarely included individuals older than 55 years of age, resulting in a lack of systematic efficacy or safety data in this population [4]. Available data suggests that efficacy of DMT might decrease with age [5, 6], albeit all comparisons across different clinical trials that vary e.g., in baseline characteristics such as a higher annualized relapse rate in younger individuals, study design, and inclusion criteria need to be interpreted with caution. One example is the efficacy data available for the sphingosine 1-phosphate receptor (S1P) modulator fingolimod compared with interferon beta-1a in the pediatric onset (aged 10–17 years, mean 15.3 years) and an adult onset MS study (aged 18–55 years, median 37 years), demonstrating consistently stronger effects in the younger population (absolute reduction for the annualized relapse rate: 45% vs. 17%) [7, 8].

With increasing age, both the innate and adaptive immune system undergo changes, termed immunosenescence [3, 4, 9]. One of the consequences of immunosenescence appears to be a reduction in the regeneration capacity of immune cells. This might especially affect T cells due to the involution of the thymus, possibly contributing to a weakening of immune competence and impaired cell mediated cancer defence [10]. Therefore, albeit age-related risks have not systematically been studied, the risk–benefit ratio of DMT might change with age not only due to a decrease in DMT efficacy, but also due to increased risks in older age groups, possibly affecting the defense against new and opportunistic pathogens, and cancer risks [1, 4].

Overall, a DMT-centered consultation of PwMS older than 55 years appears inadequate, and studies that focus on age-group specific needs are warranted. Thus, the objective of this study was to analyze the PwMS population above the age of 55 years in the German Multiple Sclerosis Registry (GMSR) concerning MS disease characteristics and DMT use, symptomatic therapy, untreated symptoms, socioeconomic status, to define the possible unmet needs and facilitate further prospective study design, and improvement of overall care.

## Materials and methods

We analyzed all patients registered in the GMSR since 2014 with a data extraction date as of 1st March 2023. Data collection and the methods for quality control of the GMSR are described in detail in Ohle et al. [11].

For this descriptive analysis, we assessed subpopulations of PwMS grouped by age as follows: above 18 to 55 years (PwMS < 55), between 55 and 64 years of age (PwMS55-64), and 65 years of age and older (PwMS ≥ 65). The variables of interest included basic characteristics, symptoms of MS, disease course, inflammatory activity, treatment and employment status, and requirement for care and assistance (for further details please see Table 1). All items were registered during the clinical visits at a documenting MS center by medical professionals filling predefined items.

**Table 1:** Overview of subject characteristics divided by age groups

Age groups (years)	PwMS < 55 (*n* = 28,313)	PwMS55-64 (*n* = 9120)	PwMS ≥ 65 (*n* = 2995)
*Subject characteristics*			
Female (%, CI)	71.0 [70.5;71.6]	70.5 [69.6; 71.4]	71.5 [69.9; 73.1]
Age (mean, CI)	41.4 [41.3;41.5]	59.2 [59.1;59.2]	70.8 [70.6;71.0]
EDSS (mean, CI)	2.73 [2.70;2.75]	4.20 [4.16;4.24]	5.26 [5.18;5.33]
Disease duration, years (mean, CI)	11.2 [11.1;11.3]	19.6 [19.4;19.9]	26.3 [25.8;26.8]
*MS disease course (%, CI)*			
CIS	1.94 [1.78; 2.11]	0.88 [0.70;1.09]	0.54 [0.31;0.88]
RRMS	84.2 [83.7;84.6]	57.9 [56.8;58.9]	33.4 [31.7; 35.2]
SPMS	8.94 [8.61;9.28]	27.8 [26.9; 28.7]	44.2 [42.4; 46.1]
PPMS	3.77 [3.54; 4.00]	12.3 [11.6; 13.0]	20.1 [18.6; 21.6]
*Employment status (%, CI)*			
Occupational invalidity	17.2 [16.8;17.7]	38.8 [37.7;39.9]	Not applicable (considering the statutory retirement age of 65 to 67 years in Germany)
Unemployment	5.45 [5.17;5.74]	3.19 [2.82;3.61]	
Education	3.32 [3.10;3.55]	0.15 [0.08;0.27]	
Full-time employment	45.3 [44.7;45.9]	25.8 [24.9;26.8]	
Part-time employment	22.2 [21.7;22.8]	19.0 [18.2;19.9]	
Retirement	1.10 [0.98;1.24]	7.03 [6.48;7.62]	
Housewife/-husband	4.65 [4.39;4.92]	5.94 [5.43;6.49]	
*Care, aids, assistive devices (%, CI)*			
Domiciliary care	19.8 [19.4–20.3]	33.6 [32.6–34.6]	52.3 [50.4–54.2]
Ambulatory care	2.7 [2.5–2.9]	8.0 [7.4–8.6]	16.7 [15.3–18.2]
Day care	0.2 [0.2–0.3]	0.5 [0.4–0.7]	2.4 [1.9–3.1]
Short term care	0.1 [0.0–0.4]	0.1 [0.1–0.2]	0.1 [0.0–0.1]
Inpatient care	0.5 [0.4–0.6]	1.5 [1.3–1.8]	3.0 [2.3–3.7]
Walking sticks	13.7 [13.3–14.1]	30.4 [29.4–31.3]	44.4 [42.6–46.2]
Wheelchair	9.2 [8.9–9.5]	22.0 [21.1–22.8]	35.2 [33.4–36.9]
Walker	10.8 [10.5–11.3]	26.6 [25.7–27.6]	45.6 [43.8–47.4]
*Reported symptoms (%, CI)*			
Gait disorder	43.4 [42.8–44.0]	73.8 [72.8–74.7]	86.4 [85.1–87.6]
Spasticity	27.1 [26.6–27.6]	47.3 [46.2–48.3]	58.2 [56.3–60.0]
Ataxia	24.6 [24.1–25.2]	38.2 [37.2–39.2]	42.9 [41.0–44.8]
Fatigue	53.0 [52.4–53.6]	61.5 [60.4–62.5]	54.0 [52.1–55.9]
Pain	25.2 [24.6–25.7]	36.1 [35.1–37.2]	43.5 [41.6–45.4]
Bladder dysfunction	29.6 [29.1–30.2]	49.2 [48.1–50.3]	60.2 [58.3–62.0]
Bowel disorder	8.2 [7.9–8.6]	14.6 [13.8–15.3]	20.0 [18.5–21.6]
Sexual dysfunction	8.5 [8.2–8.9]	13.4 [12.5–14.3]	11.4 [9.9–13.0]
Cognitive impairment	27.6 [27.0–28.1]	34.8 [33.7–35.8]	33.7 [31.9–35.5]
Depression	23.2 [22.7–23.7]	27.5 [26.5–28.5]	23.4 [21.8–25.1]
Ocular motor dysfunction	11.5 [11.1–11.9]	13.4 [12.7–14.2]	14.1 [12.8–15.5]
Dysarthria	5.5 [5.2–5.8]	8.4 [7.8–9.0]	10.9 [9.7–12.1]
Dysphagia	3.2 [3.0–3.4]	5.6 [5.2–6.2]	8.2 [7.2–9.3]
Epilepsy	1.5 [1.4–1.7]	1.7 [1.4–2.0]	2.6 [2.1–3.3]
*Surrogates of MS disease activity*			
Relapse in the last year^1^ (%, CI)	0.13 [0.13;0.14]	0.07 [0.07;0.08]	0.08 [0.07;0.09]
MRI available in the last year (%, CI)	49.3 [48.7;49.9]	40.6 [39.6;41.6]	31.3 [29.6;32.9]
MRI activity^2^ (%, CI)	23.8 [23.1;24.5]	15.1 [13.9;16.3]	15.1 [12.8;17.5]

PwMS < 55: persons with MS < 55 years, PwMS55-64: persons with MS aged 55–64, PwMS ≥ 65: persons with MS ≥ 65 years. RRMS relapsing–remitting multiple sclerosis, PPMS primary progressive multiple sclerosis, SPMS secondary progressive multiple sclerosis, CIS clinically isolated syndrome.^1^one or more clinical relapse within the preceding year,^2^ MRI activity as new or enlarging T2 lesions, or GD enhancing lesions as documented during the last visit and compared to the preceding visit

**Fig. 1:**
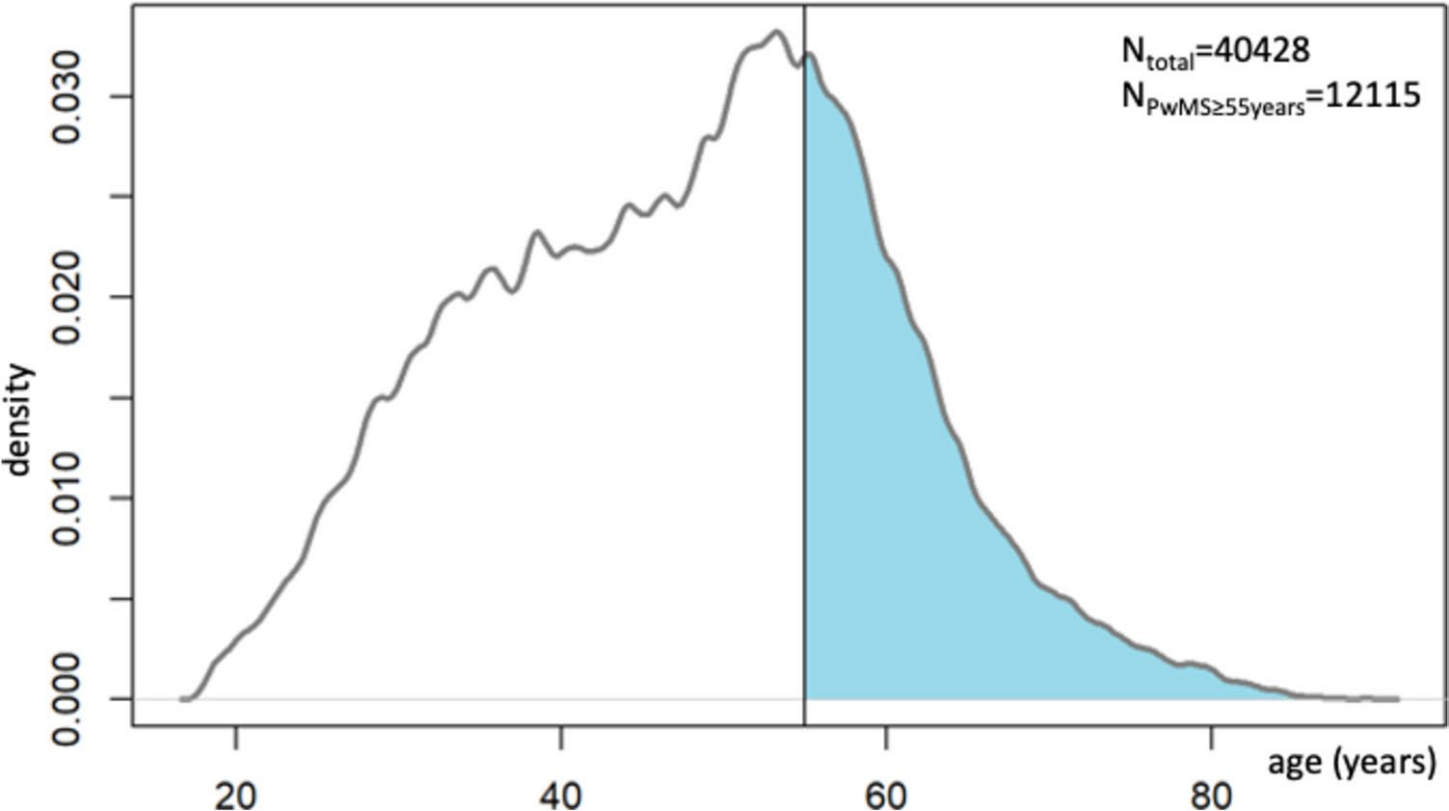
Age distribution in the total German MS registry population of 40,428 individuals. PwMs≥55 years = number of persons with MS aged 55 years or older

Comparisons between groups were analyzed at the last follow-up visit of each patient. Statistical analyses included descriptive statistics including percentages, means and standard deviations as indicated along with 95% confidence intervals (CI). We used Clopper-Pearson CI for proportions, while for metric variables we used the asymptotic z-score CI. Analyses were performed and Fig. 1 created using R v4.2.3 using the packages compare Groups 4.6.0.

### Ethics approval

The registration of the GMSR proceeded at the German Registry for Clinical Trials (Deutsches Register Klinischer Studien [DRKS]; No. DRKS00011257). The initial ethics vote was approved by University of Würzburg’s institutional review board (permit No. 142/12).

## Results

### Basic characteristics

The subject characteristics are shown in Table 1. The study population of 40,428 PwMS (Fig. 1) subsets into 70% PwMS < 55 (*n* = 28,313), 22.6% PwMS55-64 (*n* = 9120), and 7.4% PwMS ≥ 65 (*n* = 2995). The female/male ratio was comparable across the age groups. PwMS < 55 had a mean EDSS of 2.7 indicating no walking restriction, while the PwMS55-64 and PwMS ≥ 65 displayed a restricted walking range (mean EDSS 4.2 and 5.3, respectively). The frequency of individuals with a relapsing–remitting disease course declined across age groups (Table 1).

### Employment data, walking assistance and need for care

The rate of occupational invalidity was 17.2% in PwMS < 55 and 38.8% for PwMS55-64. The full employment rate was 45.3% for PwMS < 55 and 25.8% of PwMS55-64 (Table 1). In comparison, in the general population in Germany in 2022, 78.0% of persons from 18–55 and 67% of persons from 55–65 years were fully employed [12].

The need for walking assistance was more frequent among PwMS of older age: walking sticks were required by 13.7%, 30.4%, and 44.4% of PwMS < 55, PwMS55-64, and PwMS ≥ 65. Any wheelchair access (not necessarily permanent use) was documented for 9.2%, 22.0%, and 35.2% PwMS < 55, PwMS55-64, and PwMS ≥ 65, respectively.

The need for care and assistance for PwMS was higher in the older age groups. Care and support by family members was reported for 19.8% of the PwMS < 55, 33.6% for PwMS55-64, and 52.3% of the PwMS ≥ 65 (Table 1). In comparison, in the general population in Germany only 0.1–1.1% of persons below the age of 55 receive care and support by family members [12].

### MS symptoms

Disease symptom load was higher in older age groups, but patterns of symptoms were similar. Gait disorder, spasticity, ataxia, pain, bladder and bowel dysfunction, dysarthria and dysphagia were among the most frequently reported symptoms, were significantly more prevalent among the older age groups, and with the highest rates in PwMS ≥ 65 (Table 1).

Ocular motor dysfunction, sexual dysfunction, fatigue, cognitive impairment, and depression also showed higher rates in the older age groups, with an equal or even higher frequency in PwMS 55–64 as compared to PwMS ≥ 65. Epilepsy rates were significantly higher in PwMS ≥ 65.

With regards to treated symptoms, only 21.8% of sexual disorders, 32.9% of patients with fatigue and 32.1% of patients with cognitive deficits were reported to be treated across all age groups, with no detailed information on the treatment applied available.

### Inflammatory MS disease activity

The frequency of a relapse reported within the last year, registered at date of presentation, was higher in PwMS < 55 with 13%, as compared with 7% or 8% for PwMS55-64, and PwMS ≥ 65, respectively.

A cranial MRI within the last year was reported to be conducted more frequently in the younger age groups, with 49.3%, 40.6%, and 31.3% of PwMS < 55, PwMS55-64, and PwMS ≥ 65, respectively.

In those, MRI activity, defined by new or enlarging T2 lesions and/or GD enhancing lesions in comparison to the preceding scan, was more frequently noted in PwMS < 55 with 23.8%, as compared with a frequency of 15.1% both in PwMS55-64 and PwMS ≥ 65 (Table 1).

### Comorbidities

Data on comorbidities were limited, recorded for only 13.2% (5317/40428) of the total registry population. Availability of comorbidity data was similar across the age groups with 13.3% (3764/28313) for PwMS < 55, 12.7% (1160/9120) for PwMS55-64, and 13.1% (393/2995) for PwMS ≥ 65.

Vascular risk factors, such as hypertension and diabetes, were more often reported in PwMS ≥ 65: hypertension with 38.4% as compared with 24.1% in PwMS55-64 and 9.2% in PwMS < 55, and type II diabetes in 1.8% as compared with below 1% in the younger age groups.

Infections were also more frequently reported in older age groups: pneumonia in 1.3% of PwMS ≥ 65, 1.4% of PwMS55-64, and 0.7% of PwMS < 55; urinary tract infections in 5.1% of PwMS ≥ 65 vs. 4.2% of PwMS55-64 and 2.2% of PwMS < 55, and herpes simplex viral infections in 7.6% of PwMS ≥ 65 vs. 5.0% of PwMS55-64, and 3.2% in PwMS < 55 (Table 2).

**Table 2:** Patients with reported International Statistical Classification of Diseases and Related Health Problems (ICD10) diagnoses

Diagnoses according to ICD10	PwMS < 55 (*n* = 3764)	PwMS55-64 (*n* = 1160)	PwMS ≥ 65 (*n* = 393)
*Vascular comorbidities*			
I10 Essential (primary) hypertension	9.2% [8.3–10.1]	24.1% [21.6–26.6]	38.4% [33.6–43.4]
I11 Hypertensive heart disease	0.1% [0.0–0.2]	0.1% [0.0–0.5]	1.0% [0.3–2.6]
E10 Type 1 diabetes mellitus	0.3% [0.2–0.6]	0.4% [0.1–1.0]	0.8% [0.2–2.2]
E11 Type 2 diabetes mellitus	0.6% [0.3–0.9]	0.7% [0.3–1.4]	1.8% [0.7–3.6]
I25 Chronic ischemic heart disease	0.1% [0.0–0.2]	0.3% [0.1–0.8]	0.5% [0.1–1.8]
I63 Cerebral infarction	0.1% [0.0–0.3]	0.4% [0.1–1.0]	1.8% [0.7–3.6]
*Infectious complications*			
J18.0 Bronchopneumonia, unspecified	0.7% [0.5–1.1]	1.4% [0.8–2.2]	1.3% [0.4–2.9]
N39 Other disorders of urinary system	2.2% [1.8–2.8]	4.2% [3.1–5.5]	5.1% [3.1–7.8]
B00 Herpesviral [herpes simplex] infections	3.2% [2.7–3.8]	5.0% [3.8–6.4]	7.6% [5.2–10.7]
B02.0 Zoster [herpes zoster]	0.2% [0.1–0.5]	0.1% [0.0–0.5]	0.5% [0.1–1.8]

*n* = 5 317 of the total registry population, only diagnoses with a frequency of at least 0.5% in one of the age groups are shown. PwMS < 55: persons with MS < 55 years, PwMS55-64: persons with MS aged 55–64, PwMS ≥ 65: persons with MS ≥ 65 years

### Disease modifying therapy

Detailed DMT data was available for a subgroup of registered patients (Table 3). At time of the last registry entry, for 70.6% of these PwMS current DMT use was reported, with 76.7%, 60.9%, and 42.6% for PwMS < 55, PwMS55-64, and PwMS ≥ 65, respectively. Moderate efficacy DMT, including interferon beta variants, glatiramer acetate, dimethyl fumarate, diroximel fumarate or teriflunomide did not show a difference in the distribution between the age groups, with 45.9% of PwMS < 55, 47.5% of PwMS55-64, and 45.7% of PwMS ≥ 65.

**Table 3:** Disease modifying therapies (DMT) given as % and 95% confidence intervals of proportions

	PwMS < 55 (*n* = 1022)	PwMS55-64 (*n* = 2730)	PwMS ≥ 65 (*n* = 713)
*Treatment status*			
DMT use at time of last registry entry	76.7 [76.2;77.2]	60.9 [59.9;62.0]	42.6 [40.7;44.4]
DMT use any time	92.2 [91.8;92.7]	88.6 [87.5;89.6]	74.3 [71.6;76.8]
*Moderate efficacy DMT*	45.9% [44.9–46.8]	47.5% [45.7–49.4]	45.7% [42.0–49.5]
Dimethylfumarat	14.2% [13.5–14.8]	9.8% [8.7–11.0]	3.8% [2.5–5.5]
Diroximelfumarat	0.5% [0.4–0.7]	0.2% [0.1–0.4]	0.3% [0.0–1.0]
Peginterferon beta-1a	2.4% [2.1–2.7]	1.9% [1.4–2.4]	2.1% [1.2–3.4]
Glatirameracetat	11.5% [10.9–12.1]	10.8% [9.6–12.0]	9.0% [7.0–11.3]
Interferon beta-1a	6.3% [5.8–6.8]	9.1% [8.1–10.3]	11.5% [9.3–14.1]
Interferon beta-1b	3.6% [3.2–3.9]	5.5% [4.6–6.4]	9.4% [7.4–11.8]
*Higher efficacy DMT*	49.9% [48.9–50.9]	38.5% [36.6–40.3]	25.4% [22.2–28.7]
Natalizumab	11.9% [11.3–12.5]	5.3% [4.5–6.3]	2.5% [1.5–4.0]
Ocrelizumab	15.6% [15.0–16.4]	14.2% [12.9–15.6]	12.3% [10.0–15.0]
Ofatumumab	1.4% [1.2–1.7]	0.8% [0.5–1.3]	0.3% [0.0–1.0]
Ozanimod	0.9% [0.7–1.1]	0.5% [0.3–0.8]	0.1% [0.0–0.8]
Ponesimod	0.1% [0.1–0.2]	0.1% [0.0–0.3]	0.1% [0.0–0.8]
Rituximab	0.9% [0.7–1.1]	1.0% [0.7–1.5]	0.8% [0.3–1.8]
Siponimod	0.8% [0.6–1.0]	2.7% [2.1–3.4]	1.8% [1.0–3.1]
Alemtuzumab	2.7% [2.4–3.0]	0.7% [0.4–1.1]	0.3% [0.0–1.0]
Cladribin	2.9% [2.6–3.2]	2.4% [1.9–3.1]	1.7% [0.9–2.9]
Fingolimod	12.5% [11.9–13.2]	10.6% [9.5–11.8]	5.3% [3.8–7.2]
Other DMT (methotrexate, daclizumab, mitoxantrone, azathioprine, repetitive steroid therapy outside of relapses, cyclophosphamide)	4.3% [3.9–4.7]	14.0% [12.7–15.4]	28.9% [25.6–32.4]

PwMS < 55: persons with MS < 55 years, PwMS55-64: persons with MS aged 55–64, PwMS ≥ 65: persons with MS ≥ 65 years

Grouped higher efficacy drugs (monoclonal antibodies (alemtuzumab, natalizumab, ocrelizumab, rituximab, ofatumumab), purine analog (cladribine), sphingosine I-phosphate receptor modulators (fingolimod, ozanimod, ponesimod)) were more frequently recorded in the younger age groups, with 49.9%, 38.5%, and 25.4% for PwMS < 55, PwMS55-64, and PwMS ≥ 65, respectively.

Looking separately at single compounds, no significant differences were noted for the use of cladribine, ocrelizumab, and rituximab across age groups. Natalizumab, ofatumumab, fingolimod, and ozanimod were more frequently reported in the younger age groups. Siponimod, registered in the EU for active SPMS only, was more frequently used in the older age groups (Table 3).

## Discussion

We analyzed data from the German MS registry (GMSR) with a focus on PwMS ≥ 55, comprising around 30% of the total GMSR population, a rate similar to what has been reported from other European MS registries [13].

Several findings confirmed the expected. Disease duration and disability as assessed by EDSS were higher. As the EDSS is largely dependent on gait, not surprisingly, walking restrictions were seen in around 74% of PwMS55-64, and 86% of PwMS ≥ 65. This also associated with a higher likelihood of loss of independence, the need for walking aids, and domiciliary care. For a majority of around 52% of PwMS ≥ 65 in our German registry population care at home by family members and friends were reported, in comparison with the general population aged between 65–70 years in Germany of whom 18.2% receive ambulatory care [14]. Higher disability associated with lower rates (only around 26%) of full-time employment in PwMS55-64, and a comparably high frequency of occupational invalidity seen in around 39%. Furthermore, highly disabling symptoms such as bowel and bladder dysfunction and cognitive impairment were witnessed at higher frequency in PwMS ≥ 55, likely impacting the conduct of an independent life.

Furthermore, PwMS ≥ 55 in our cohort showed less signs of disease activity as reflected by clinical relapses and inflammatory MRI activity. Fitting literature and the general findings in an aging MS population [15], this associated with a higher frequency of progressive forms of MS noted in PwMS > 55, and a lower frequency of DMT use.

Interestingly, and not necessarily expected, in particular higher efficacy DMT were less frequently applied in PwMS > 55, with a rate of 38.5% in PwMS55-64 and 25.4% in PwMS ≥ 65 in comparison to 59.9% of PwMS < 55. This finding might be explained by a lack of efficacy data of most of the DMT in older age groups, and safety concerns. Not only for S1P, as mentioned in the introduction, but also for anti-CD20 therapies, stronger effects were shown in the younger and less progressed relapsing MS (RMS) study populations (mean age 37.2 years) compared to the older population with progressive MS (PMS, mean age 44.7 years) [16]. The witnessed moderate positive overall effect of ocrelizumab in PPMS was driven by individuals early in the disease course: aged below 45 years, and with MRI signs of inflammation [17]. Similar data are available for DMT that are used also for later stages of RMS with clinical progression, including cladribine [18], ocrelizumab [19], rituximab [20] and ofatumumab [21], or were tested specifically in SPMS (siponimod) [22]: the information available from these phase II/III studies suggest efficacy in particular if these drugs were applied at a relatively young age (below 45 years) and when signs of inflammatory activity were present (MRI activity or clinical relapses).

Regarding safety aspects, we noted a higher frequency of vascular comorbidities, such as hypertension and type 2 diabetes, in the older GMSR population as compared to the PwMS < 55. As vascular risks increase with age, this may be expected, but needs to be considered e.g., also when MRI is analyzed, as not all new or enlarging T2 lesions might reflect inflammatory MS disease activity. Furthermore, a higher rate of pneumonia, urinary tract infections and herpes viral infections were noted in PwMS ≥ 55. As one of the limitations to our study, data on comorbidities such as infections were restricted to a smaller subset of around 13% of the total GMSR cohort. Nevertheless, the observation in our cohort fits with concerns that older PwMS might be at higher risks, in particular when treated with immunosuppressive DMT, including opportunistic infections and malignancy [23], possibly cumulating with multiple previous DMT [24, 25]. Albeit data is scarce, higher rates of herpes viral infections (varicella zoster reactivation or herpes simplex virus activation) are noted with natalizumab, fingolimod, alemtuzumab and cladribine [4], and in particular VZV reactivation are known to occur at a higher frequency with increasing age. Older age possibly increases the risk also of opportunistic infections such as progressive multifocal leukoencephalopathy (PML) during therapy with fingolimod, and possibly increases mortality in natalizumab-associated PML cases [4]. Furthermore, hypogammaglobulinemia is a known adverse effect of B-cell depleting therapies. Comparing data from the older aged ocrelizumab PPMS trial with data from the ocrelizumab RMS trials, it appears that in the PPMS population the proportion of individuals with IgG and IgM levels below the lower limit of normal is higher, possibly linked with an increase in serious infections in the older population [26]. Most frequent infections were common infections, such as respiratory tract infections and cystitis [27]. During the COVID-19 pandemics, it was noted that PwMS were not generally at increased risk for a severe COVID-19 [28]. However, a higher grade of disability, vascular comorbidity, progressive MS, B-cell depleting therapy, and advanced age were associated with a more severe COVID-19 disease course [29]. Larger real-world datasets with accurate reporting of safety and DMT efficacy data should be analyzed with regards to the question whether the continuation of higher efficacy DMT overall remain to display a positive risk–benefit ratio in PwMS ≥ 55. Thus far, only a smaller set of studies started to assess treatment interruption in patients that are stable clinically and on MRI. In a recently published study restricted to PwMS ≥ 55, non-inferiority of stopping MS therapy (with the majority of PwMS treated with moderate efficacy DMT) could not be demonstrated for a combined clinical and MRI endpoint, but risk of recurrence of clinical disease activity was low during the observational period of the study [1]. Furthermore, the ‘DOT-MS study’ that also included younger PwMS had to be discontinued because disease activity reoccurred in 9 out of 45 patients after discontinuation of DMT in comparison to none in the ongoing DMT group [30].

In summary, the data from the GMSR, a large national registry, shows that around 30% of the MS population is above the age of 55 years. In this subpopulation, MS related disability, symptom load and the need for assistance and care differs from the younger age groups. Furthermore, lower signs of MS disease activity both regarding relapses and inflammatory activity on MRI can be noted, associating with a lower frequency of use of any DMT, and in particular of higher efficacy DMT. The rate of prescription of immunosuppressive DMT appears to decrease with age, while the relevance of symptomatic therapy, care, and assistance for PwMS, their caregivers and socio-economical support increases. We conclude that studies that specifically focus on PwMS ≥ 55, who have multi-layered symptoms and complex needs, a higher grade of dependency and limited resources, are needed. Besides treatment interruption studies in PwMS such as the DISCOMS study [1], interventional prospective studies with broader offers to the vulnerable group of PwMS ≥ 55 are needed. As a first step in the administrative district of Cologne, Germany, a phase II clinical study exploring the value of a case and care management with cross-sectoral coordination of services for severe forms of MS, including a larger subpopulation of PwMS ≥ 55, was completed (COCOS-MS trial [31, 32]), with results expected in 2024. The findings of this study might complement the registry data presented here, helping to design a lager multicenter interventional study aiming at improving the life of PwMS > 55.

## Data Availability

Anonymized data will be made available on request for any qualified investigator under the terms of the registries’ usage and access guidelines and subject to the informed consent of the patients.
